# Effects of School Violence on Depression and Suicidal Ideation in Multicultural Adolescents

**Published:** 2019-02

**Authors:** Eunmi LEE

**Affiliations:** Department of Nursing, Hoseo University, Asan, Korea

**Keywords:** School violence, Depression, Suicidal ideation, Multicultural adolescents

## Abstract

**Background:**

This study investigated how experiencing school violence affects depression and suicidal ideation in multicultural adolescents.

**Methods:**

Overall, 63444 adolescents from grades 7–12 (ages 12–18 yr) who participated in the 11th Korea Youth Risk Behavior Web-based Survey (KYRBS-XI) were enrolled from Jun 1 to Jun 30, 2015. Adolescents with at least one parent of non-Korean nationality were classified as multicultural adolescents. A logistic regression analysis of depression and suicidal ideation on the experience of violence was conducted with age, gender, city size, academic achievement, economic status, and level of stress as control variables.

**Results:**

Multicultural adolescents were found to have low grades, low economic status, come from smaller cities, and experience more school violence and stress compared to non-multicultural adolescents. Multicultural adolescents who experienced school violence were 5.41 times (95% CI 3.22, 9.11) more likely to be depressed than those who did not experience violence, whereas, non-multicultural adolescents were 3.75 times (95% CI 3.34, 4.20) more likely to be depressed. The effect of school violence on suicidal ideation was also stronger for multicultural adolescents. Multicultural adolescents who experienced school violence were 7.78 times (95% CI 4.34, 13.96) more likely to have suicidal ideation than those who did not experience violence, while non-multicultural adolescents were 4.17 times (95% CI 3.64, 4.78) more likely to have suicidal ideation.

**Conclusion:**

Adolescents who experience school violence more frequently showed a higher risk for depression and suicidal ideation, and the effect of school violence on depression and suicidal ideation was relatively stronger in multicultural adolescents.

## Introduction

Multicultural families have become an integral part of society with an increase in the number of international marriages and the influx of foreign workers. The demand for multicultural families to go beyond the basic settlement stage of initial formation towards a more socially and economically integrated settlement is increasing (1). According to the National Survey of Multicultural Families 2015, there were 278,036 multicultural households in Korea, and 82,476 adolescents were from multicultural families. This shows a 24.0% increase from the 2012 survey (66,536 adolescents), a trend that reflects the increase in the number of multicultural families.

With an increase in the number of international marriages and the birth and growth of children in multicultural families, children and adolescents reach their potential as important members of society. However, in reality, adolescents from multicultural families discontinue their studies due to difficulties in learning activities, relationships with peers and teachers, and family circumstances, more often than the general population (2). As the number of single-parent and dual-income families is increasing, school violence is also increasing owing to the weakened caregiving functions of families. The growing influence of media violence and diminished functions of schools and teachers are other causes of school violence.

There is growing concern over the need for societal measures to be established in response to school violence being raised as a serious juvenile issue. Particularly for multicultural adolescents with distinct physical characteristics or cultural backgrounds, differences in outward appearance can lead to social exclusion, and school adjustment tends to be low when adolescents are aware of these differences (3). The proportion of multi-cultural adolescents experienced school violence is 5.0%; 5.3% males and 4.6% females. The rate of school violence decreases with an increase in the age of the students (2). When compared to adolescents from typical families, 44.3% of multi-cultural adolescents experienced physical violence and fights, which is higher than the frequency for non-multicultural adolescents (34.2%) (4). The resultant stress in adolescents increases the risk of depression (5) and suicide (6), as well as the risk of deviant behaviors such as smoking and alcohol consumption (7). Multicultural adolescents experience not only the difficulties of puberty but also experience adjustment stress that stems from cultural and ethnic differences since culture can intensify the stress experience compared to general kinds of stress (8, 9). Gender is another aspect considered when evaluating multicultural adolescents. Although existing studies have examined the mental health of multicultural adolescents (10, 11), they offer only a piecemeal view by limiting the scope to multicultural adolescents. No study has explored the relative strength of the relationship among violence, depression, and suicide by making cross-comparisons between multicultural and nonmulticultural adolescents. Thus, the present study examined and compared the effects of experiencing school violence on depression and suicide in multicultural adolescents and non-multicultural adolescents.

## Materials and Methods

Multicultural adolescents were identified as having at least one parent of non-Korean nationality. Raw data were obtained from the 11th Korea Youth Risk Behavior Web-based Survey (KYRBS-**XI**), conducted from Jun 1 to Jun 30, 2015, to investigate the health behaviors of Korean adolescents from grades 7–12 (aged 12–18 yr) (12). The participants were middle and high school students from across Korea and were selected in Apr 2014 from a sampling frame based on school type and district area. The 11th (2015) survey included 68,043 students from 797 schools out of a total of 800 schools including 400 middle schools and 400 high schools, and data from 63,444 students were used for the present study after excluding those without parents. The experience of school violence was considered an experience of hospital treatment due to school violence.

The KYRBS-XI consists of 125 items across 14 domains including tobacco use, alcohol use, physical activity, dietary behaviors, obesity and weight control, mental health, unintentional injury behaviors, oral health, personal hygiene, sexual behaviors, atopy and asthma, internet addiction, health equity, violence, and subjective health (12). The present study used variables relevant to multicultural family, experience of violence leading to hospital treatment, mental health, and general characteristics.

Stress, depression, and suicidal ideation were variables used in the mental health category. Age, gender, city size, academic achievement, and economic status were used for general characteristics. The study was approved by the university’s Institutional Review Board (no. 1041231-150616-HR-028-01).

### Statistical analysis

To compare the general characteristics of multicultural and non-multicultural adolescents, a cross-tabulation analysis and the chi-square test of homogeneity were used by SAS (9.2). As age and stress level are quantitative variables, they were analyzed using the independent samples t-test. To examine the relationship between adolescents’ experience of school violence and multi-culturalism, holding general characteristics constant, the Cochran-Mantel-Haenszel (CMH) test was conducted (13). In addition, to analyze whether the degree of association between experience of school violence and multiculturalism (odds ratio) differs across general characteristics, the Breslow-Day test was used. Of the general characteristics, age and stress level were analyzed as main effects, interaction effects, and confounding effects in the linear model. Lastly, logistic regression was performed for both adolescent groups with experience of school violence as an independent variable, depression and suicidal ideation defined as dependent variables, and general characteristics and stress as control variables. For the logistic regression analysis, 95% confidence intervals were constructed for the odds ratio estimates, and the Wald statistic was used to test for homogeneity in order to compare multicultural and non-multicultural adolescents.

To analyze the effect of experiencing school violence on depression depending on adolescents’ multiculturalism, a logistic regression analysis was performed to examine the odds ratios in the four models. In model 1, the independent variable was the experience of school violence and the dependent variable was depression/suicidal ideation. In model 2, stress was added to the first model as a control variable. In model 3, general characteristics like gender, age, academic achievement, economic status, and city size were added to the first model as control variables. In model 4 (saturated model), stress and general characteristics, gender, age, academic achievement, economic status, and city size, were added to the first model as control variables. Among these models, a model with significant and control variables was adopted as the final model.

## Results

Of the 68,043 participants in the KYRBS-**XI**, 63,444 remained after excluding those with both parents absent. Of this, 795 (1.3%) adolescents were from multicultural families, and 62,649 were not. The number of adolescents who experienced school violence was 1,271 (2.0%), and 62,173 had no such experience.

The distribution of the general characteristics in the multicultural and non-multicultural adolescent groups showed statistically significant differences between the two groups, except for gender. For academic achievement and economic status, multicultural adolescents were highly distributed in the average and low range, showing lower figures in both indicators compared to nonmulticultural adolescents. Regarding the size of the cities in which the students resided, a disproportionate number of multicultural adolescents resided in the county areas whereas nonmulticultural adolescents resided in the metropolitan areas. There was a statistically significant difference in the mean ages of the two groups, with the multicultural adolescents being 0.23 yr younger than the non-multicultural adolescents. A large difference was seen in the domain of school violence with 10% of multicultural adolescents having experienced school violence compared to 1.9% for non-multicultural adolescents. That is, more multicultural adolescents were shown to be victims of violence than nonmulticultural adolescents. Multicultural adolescents showed a statistically significant difference in terms of high levels of perceived stress (Table 1).

**Table 1:** Descriptive Statistics of Multicultural and Non-multicultural Adolescents

**Variable**		**Non-multicultural adolescents (%)**	**Multicultural adolescents (%)**	**χ^2^, t**	***P*-value**
Gender	Male	52.1	53.7	.705	.401
	Female	47.9	46.3		
Academic achievement	Very high	12.9	10.9	45.128	<.001
	High	25.7	20.8		
	Average	28.2	25.0		
	Low	23.2	26.6		
	Very low	10.1	16.8		
Economic status	Very rich	9.4	8.6	211.793	<.001
	Rich	28.5	15.9		
	Average	47.4	45.9		
	Poor	12.2	20.1		
	Very poor	2.5	9.5		
City size	Metropolis	44.5	36.0	114.190	<.001
	Small and medium	48.6	46.9		
	County	6.9	17.1		
Experience of school violence	No	98.1	90.0	228.524	<.001
	Yes	1.9	10.0		
Age (12–18, mean±SD)		14.94±1.74	14.71±1.73	3.660	<.001
Stress (0–5, mean±SD)		3.18±0.94	3.29±1.02	2.880	.004

Table 2 shows the association between adolescents’ multiculturalism and experience of school violence after removing the effects of general characteristics. Statistically significant differences were found for all variables (compared using the CMH test). Examining whether the degree of association (odds ratio) between multiculturalism and experience of school violence differs for each item of the general characteristics revealed statistically significant differences for all variables (compared using the Breslow-Day test).

**Table 2:** Experience of school violence according to sociological characteristics among multicultural and non-multicultural adolescents

***Variable***		***Non-multicultural adolescents (%)***	***Multicultural adolescents (%)***	***Total (%)***	***CMH Test***		***Breslow-Day Test***	
					***χ^2^***	***P-value***	***χ^2^***	***P-value***
Total		1.9	10.0	2.0				
Gender	Male	2.8	11.3	2.9	224.866	<.001	7.157	.008
	Female	1.0	8.6	1.1				
Academic achievement	Very high	2.6	24.9	2.8	210.635	<.001	12.334	.015
	High	1.4	3.9	1.4				
	Average	1.6	7.3	1.6				
	Low	2.1	10.0	2.2				
	Very low	3.2	12.0	3.3				
Economic status	Very rich	3.6	28.7	3.9	173.976	<.001	12.654	.013
	Rich	1.6	4.8	1.6				
	Average	1.6	4.3	1.6				
	Poor	2.1	11.5	2.3				
	Very poor	6.2	26.4	7.1				
City size	Metropolis	1.9	12.2	2.8	216.550	<.001	11.575	.003
	Small and medium	1.9	10.6	2.0				
	County	2.7	3.8	1.9				

Gender differences were found among multicultural adolescents, with males experiencing more school violence than females (males 11.3%, females 8.6%). When students with very high or very low academic achievement were multicultural adolescents, the proportion experiencing school violence was higher (24.9% and 12.0%, respectively). Multicultural adolescents with very high or very low economic status had higher rates of experiencing school violence (28.7% and 26.4%, respectively). In other words, multicultural adolescents were more likely to be victims of school violence when their academic achievement or economic status was either very high or very low.

Examining the level of stress depending on adolescents’ experience of school violence and multi-culturalism revealed that the interaction between experience of school violence and multiculturalism was statistically significant, but not a large effect. Examining the age differences depending on adolescents’ multiculturalism and experience of school violence showed a statistically significant association between age and experience of school violence, as well as multiculturalism. In other words, age differences in the experience of school violence were found between multicultural and non-multicultural adolescents, with multi-cultural adolescents who experienced school violence being relatively older than adolescents from non-multicultural families (Table 3).

**Table 3:** Differences in age and stress based on the experience of school violence among multicultural and non-multicultural adolescents

***Variable***	***Experience of violence***	***Non- multicultural adolescents***	***Multicultural adolescents***	***Total***	***P-value***
Stress (0∼5)	Yes	3.43±1.14	3.20±1.40	3.42±1.16	a) .331 b) .537 c) .002
	No	3.18±0.94	3.33±0.97	3.18±0.94	
Age (12∼18)	Yes	14.71±1.70	15.62±1.83	14.73±1.71	a) .029 b) .041 c) <.001
	No	14.97±1.72	14.69±1.70	14.96±1.72	

a) Test about the experience of school violence

b) Test on multicultural adolescents

c) Test about the interaction between experience of school violence and multicultural adolescents

The effect of experiencing school violence on depression was statistically significant and relatively stronger in multicultural adolescents compared to non-multicultural adolescents. The difference between multicultural and nonmulticultural adolescents was statistically significant (compared by test of homogeneity using the Wald statistic) (w=5.380, *P*=.020). Depression in multicultural adolescents who experienced school violence was 5.41 times (95% CI 3.22, 9.11) higher than those who did not, but, in nonmulticultural adolescents, it was 3.75 higher (95% CI 3.34, 4.20), and this difference was statistically significant. That is, the effect of experiencing school violence on depression was relatively stronger for multicultural adolescents (Table 4). The effect of the experience of school violence on suicidal ideation was statistically significant and relatively stronger in multicultural adolescents compared to non-multicultural adolescents. In the odds ratio test of homogeneity, the difference was statistically significant for the reduced model (model 1; w=10.693, *P*=.001) and the model controlling for stress (model 2; w=4.906, *P*=.027). In the model that controlled for stress, suicidal ideation was 7.78 times (95% CI 4.34, 13.96) higher in multicultural adolescents who experienced school violence than those who did not, whereas, in adolescents from non-multicultural families, it was 4.17 times (95% CI 3.64, 4.78) higher, and this difference was statistically significant.

**Table 4:** Depression and Suicidal Ideation due to the experience of school violence

***Variable***			***Odds Ratio (95% CI)***
Depression (Model 1)	Multicultural adolescents (Odds ratio (95% CI))		5.414 (3.216, 9.112)
	Non-multicultural adolescents (Odds ratio (95% CI))		3.745 (3.342, 4.197)
	Homogeneity test	Odds ratio (95% CI)	1.220 (1.031, 1.444)
		Wald (*P*-value )	5.380 (.020)
Suicidal Ideation (Model 2)	Multicultural adolescents		7.779 (4.335, 13.957)
	Stress (adjusted effect)		3.153 (3.054, 3.255)
	Non-multicultural adolescents		4.172 (3.640, 4.781)
	Stress (adjusted effect)		2.076 (1.668, 2.583)
	Homogeneity test	Odds ratio (95% CI)	1.282 (1.029, 1.597)
		Wald (*P*-value )	4.906 (.027)

Model 1: Simple model.

Model 2: Adjusted for the stress index.

That is, the results show that the effect of experiencing school violence on suicidal ideation is relatively higher in multicultural adolescents (Table 4).

## Discussion

The present study investigated whether the effects of experiencing school violence on depression and suicidal ideation differ between multi-cultural and non-multicultural adolescents. Multi-cultural adolescents in Korea, compared to their non-multicultural counterparts, were found to have lower academic achievement and economic status, live in smaller cities, experience school violence and stress more frequently, and display poor quality of life and living environment. These findings are consistent with existing research (2, 4, 10). The risk of being exposed to school violence decreases as age increases; as seen in 11.1% elementary school students, 10.0% middle school students, and 4.2% high school students who experienced school violence for the first time (14). Our results showed that, for multicultural adolescents, the experience of school violence is more frequent if the students are male, younger, and have a low family income. This finding was consistent with existing research (2, 14). However, the finding that students from high-income families frequently experience school violence suggests that an approach that focuses solely on the low income multicultural adolescents may lead to biased results. School violence can be reduced through prevention education, counseling, as well as various intervention programs in schools (15, 16). Thus, the Korean government needs to implement various school violence prevention programs and interventions in collaboration with the school and local community to reduce stress in multicultural adolescents and promote successful adjustment and functioning.

In 2015, adolescent suicide was 7.2 per 100,000 population, and the second-largest number of transportation accidents was 4.0 per 100,000 population (17). The leading cause of death in adolescents in Korea from 2006 to 2015 was suicide (17), and stress and depression have been pointed out as the main causes of suicide (10). In particular, research examining adolescents’ exposure to violence, as it is related to emotional problems, has found that exposure to violence leads to posttraumatic stress disorder and depression, and is related to a high prevalence of depression and suicidal ideation in adolescents (18–20). In addition, multicultural adolescents experience violence more frequently compared to nonmulticultural adolescents, and that low family income is related to an increase in depression (2, 21). This supports the present finding that multi-cultural adolescents are subject to a low quality of life and poor living conditions, and that experience of violence has a relatively strong effect on depression and suicidal ideation under such conditions.

However, this study is limited in that the KYRBS-XI is a cross-sectional study. In addition, this was a secondary analysis and restructuring of data collected in a large-scale survey by a government agency. Therefore, further research on the degree of exposure to violence, type of violence, and perpetrators of violence, will yield more useful data for building programs, for multicultural adolescents designed to prevent school violence, and provide care for victims of violence. As the survey was conducted on an adolescent sample in Korea, the results of the present study cannot be generalized to the whole multicultural adolescent population. However, the data can serve as groundwork for perceiving multicultural adolescents as important members of society and providing policy-level support for them. Since the existing research on the severity of school violence, and the prevention and care programs being implemented target non-multicultural adolescents, there is a need for policies and programs for multicultural adolescents. Qualitative research providing data that are more detailed and research on developing prevention and depression intervention programs are needed.

## Conclusion

The experience of school violence in adolescence can lead to depression and suicidal ideation. In particular, this effect was relatively stronger in multicultural adolescents, compared to nonmulticultural adolescents. Gender, age, academic achievement, income, and place of residence were all factors that influenced the experience of school violence in multicultural adolescents. Therefore, national policies and health education programs need to be created to reduce depression and suicidal ideation in adolescents who experience school violence, especially those coming from multicultural families.

## Ethical considerations

Ethical issues (plagiarism, informed consent, misconduct, data fabrication and/or falsification, double publication and/or submission, redundancy, etc.) have been considered by the author.
